# Procedures Relative to the Single Care of Insane Persons

**Published:** 1900-07-21

**Authors:** F. St. John Bullen


### Hospital Clinics and Medical Progress.

### "PROCEDURES RELATIVE TO THE SINGLE CARE OF INSANE PERSONS."

By F. St. John Bullen, M.R.C.S.

Providing that suitable care and control be exercised an insane person may be kept at home by relatives, may reside with friends, or in a private family or charitable establishment without any legal formalities; but persons not being relatives must not keep, board, or lodge any insane person without proper legal surveillance, if they receive any pecuniary recompense or derive any profit thereby. Moreover, even when a patient is taken charge of without payment, and without legal formalities, the Commissioners in Lunacy on becoming cognisant of the case may call for the Lord Chancellor's inquiry into the circumstances. In the event of the charge of an insane person being assumed by relatives, such interference would only be made on the supposition of neglect, want of essential control, cruelty, or suspicion of wrongful application of the lunatic's property.

Any fit and proper person may take charge and control of an insane person, provided this one be duly committed to their special care under the requisite legal process, and the duties and functions of the person in charge differ very little whether he be a medical man or no.

Only under exceptional circumstances can more than one certified lunatic be received at a time into a private house; and where borderland cases are kept there together with such certified patient, the Commissioners would probably institute a very searching inquiry into the mental condition of these, and the fitness of their society for the insane person. It is very important that where one or more so-called borderland cases are received into a private house, especially if a certificated case of insanity be there also, that no question of alleged lunacy can arise. If the proprietor of the house be a medical man he should ask himself if it would be possible for him to certify any of these residents, and if in doubt should protect himself by a second and expert opinion. If not a medical man, skilled advice should certainly be taken in any suspicious case. The Commissioners naturally show much care in investigating the surroundings of a certified lunatic who is supposed to be benefitting by the special advantages of single care, and demand that the environment be as favourable as possible, whether in the shape of persons or things.

The borderland cases most often giving rise to trouble are those of hysteria with exacerbations into mania or melancholia, early or chronic and mild cases of delusional insanity, kindly called eccentrics or cranks, imbeciles and demented persons. Such cases are not at all infrequently kept uncertified for a much longer period than is justifiable. Although the surroundings of a certificated lunatic must be adjusted to the pecuniary recompense, it will be necessary that in any case moderate comfort, cleanliness, and sufficiently good dietary be evident, that the surroundings be adapted to insure the safety of the patient, and that proper attendance and supervision be provided. Arrangements for exercise and attempts to amuse and divert the patient must be evidenced, and the general disposition of the house and the inmates with whom the patient is associated must be shown to be satisfactory. All such conditions are under the surveillance of the patient's medical attendant, and the Commissioners or representative authorities. The Commissioners have power to inquire into the money received on account of the patient and its expenditure. The object of the law has been, so far as possible, to guard against complicity between any of the persons who are engaged in depriving the patient of his liberty; and as the person assuming charge of the insane is debarred, under penalty, from doing so unless the documents authorising detention are in material points valid, attention is here drawn to certain prohibitions made in furtherance of the above object. Therefore no relationship by blood or marriage, nor any business connection, may exist between the two medical men who sign the certificates, nor between either of them and the petitioner, the future medical attendant, or the person to have charge of the patient. The judicial authority must not have any relationship or business connection with either the petitioner, the lunatic, or wife or husband of the latter. But relationships between either petitioner or justice and the medical attendant do not transgress, nor do step-relationships between any parties. And the person having charge of the patient is forbidden to appoint a partner, assistant, son, father, or brother as medical attendant. Other relationships are not mentioned, but it needs to be remembered that the Commissioners have always the power to change the medical attendant of a single patient, and other existing relationships, though not transgressing the Act, might call for their intervention. Lastly, no prohibition is made against business or blood relationship between the medical certifiers and the judicial authority.

Although it is seldom that the justice who makes the order for the detention of the patient does not first see the latter, this eventuality is provided for by giving the patient the right of interviewing another justice having jurisdiction in the place where the lunatic is confined. This right must be made known to him within 24 hours, exclusive of the day of reception, by a written notice, and he may exercise it at any time within seven days after its receipt.

The person having charge of the patient must forward unopened all letters addressed by the patient to the Commissioners in Lunacy, Lord Chancellor or his visitors, Secretary of State, Judge in Lunacy, the person signing reception order or petition, whatever be their nature; other letters may be dealt with at discretion. So also all visitors sanctioned by such authorities must be allowed to interview the patient, but under no conditions must the signature of the patient be obtained by any visitor for the transaction of any business. The person in charge will, therefore, be within his rights in refusing any visitor, not authorised as above, an interview with the patient, if he considers that such might be prejudicial in its effects. In this case it is necessary to enter a note in the Medical Journal stating the grounds for refusal.

The petitioner or his deputy must visit the patient at least once in every six months, and the name of the visitor, and date of the visit, must be noted in the Medical Journal.

The Commissioners or their representatives will visit one or more times a year, and besides this, one of them, or a deputy, may at any time interview the patient and inquire into all circumstances in connection with his maintenance. In the case of a Chancery lunatic, the Chancellor's visitors will see the patient four times a year during the two years following that of the inquisition, and the Patients' Committee will visit not less often than twice yearly, and will frame a report as to the patient's condition, which needs the countersignature of the medical attendant.

